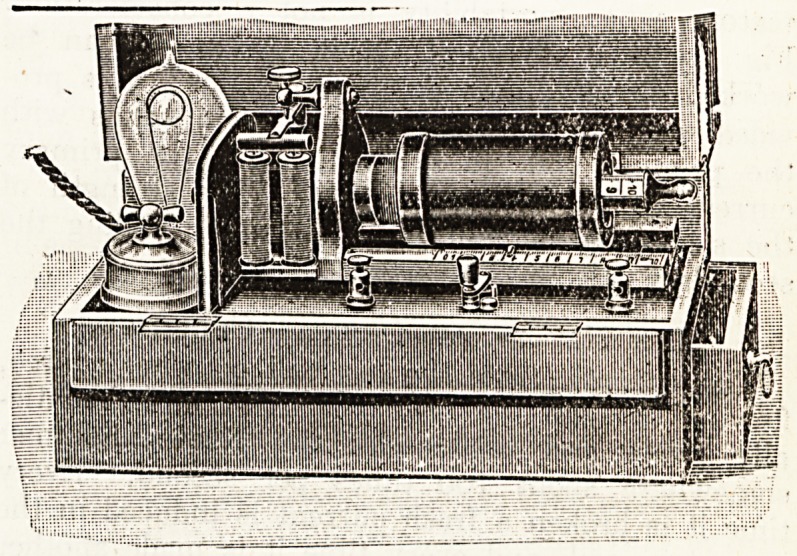# Faradic Electricity

**Published:** 1913-03-01

**Authors:** Alfred C. Norman

**Affiliations:** House Surgeon at Durham County Eye Infirmary.


### ELECTRICITY IN MODERN MEDICINE.*

### XXVI. Faradic Electricity.

By ALFRED C. NORMAN, M.D. Edin., House Surgeon at Durham County Eye Infirmary.

Electric currents produced by induction coils are known in medical practice as "Faradic," in memory of their great discoverer—Michael Faraday. These currents are more frequently applied to the human body than all other methods of electrical treatment taken together, simply because we have in the faradic coil (or "medical shocking coil," as it is sometimes termed) a simple and inexpensive means of producing currents of sufficient voltage to overcome the high resistance of the human skin.

In the section on galvanic electricity it was pointed out that to force an appreciable current through the skin it was necessary to employ a pressure of from 10 to 50 volts—depending upon the form of the electrode and the amount of moisture in the skin—and that to obtain this voltage from a galvanic battery we should have to connect a number of cells together in series. Now, by means of a faradic coil (which is simply a small induction coil) it is an easy matter to transform the current from a single dry cell to any voltage that may be required for electrical treatment. Of course, the total number of watts (volts multiplied by amperes) obtained from the coil will not be greater than the total number furnished by the dry cell. We shall be simply obtaining an increased voltage at the expense of a diminished amperage, and this is exactly what we require in medical practice, for it is never necessary to apply more than a fraction of an ampere to the human body.

If an induction coil could be made to furnish a *continuous* current, as well as an alternating, the troubles of the beginner would be at an end; but, unfortunately, *all* currents generated by induction coils, or static transformers, must necessarily be *alternating* and *interrupted* in character. Now, alternating currents have a very different action upon muscle and nerve from that of continuous currents; hence for treating many forms of nervous disease and for muscle testing it is necessary to use both kinds of current, with a consequent multiplication of apparatus.

### Medical Induction Coils.

If the reader will refer to page 653 of The Hospital for March 30, 1912, he will find the principle of the induction coil expounded at some length, so that it will be only necessary now to consider special modifications required for purposes of medical treatment.

The medical induction coil is, of course, much smaller than the *x*-ray coil; in fact it measures but a few inches in length and would not send a spark across an air-gap of $\frac{1}{8}$ of an inch. The interrupter is permanently attached to the coil, and consists of a Neef's hammer, which oscillates between two contacts as a result of magnetic attraction, and thus makes and breaks the primary circuit.

There is no condenser; the secondary coil is made much shorter in proportion, and the iron core much smaller than in the case of the *x*-ray coil.

*The rate of interruption* can be varied within certain limits by adjusting the contact screw of the Neef's hammer, thus altering the tension of the spring and the distance through which it travels. On some coils the rate of interruption is varied by means of a sliding weight which can be moved along a vertical arm, thus exerting a pendulum-like action on the oscillations of the contact maker.

*The strength of the current* obtained from the secondary coil may be controlled by various methods. In some coils the iron core is made to slide into or out of the secondary coil at will, the current, of course, being strongest when the core is pushed right home. In other coils there is a brass tube which can be pushed over the iron core so as to shield the coil from the magnetic lines of force set up by the iron core; in this case, obviously, the current is strongest when the brass tube is completely withdrawn, and weakest when it is pushed right home. A third method is to utilise a crank and a series of studs in order to throw into action a larger or a smaller number of turns of secondary wire as may be required, but the resulting variations in current strength are too abrupt to be satisfactory. The most satisfactory method of varying the current strength, and the method always adopted on the best coils, is to make the secondary coil slide along on a sort of sledge in such a way that it can be made to completely surround the primary or be withdrawn from it at will. The more the secondary coil overlaps the primary the stronger will be the

* Previous articles appeared on Nov. 11, 25, Dec. 9, 30, 1911; Jan. 13, 27, Feb. 17, March 9, 30, April 20, May 4, 25, June 8, July 6, Aug. 3, 17, 31, Sept. 28, Oct. 12, 26, Nov. 9, 30, Dec. 14, 1912; Jan. 11, and Feb. 1.

secondary current, and if there is a millimetre scale on the base of the coil it is always possible to compare results in muscle testing, for instance, by noting the position of the sledge with regard to the primary coil. There is no simple and reliable method of directly measuring the current strength from a medical coil; various faradimeters have been devised for the purpose, but they are complicated and only approximately correct. Every worker must get thoroughly used to his own coil, and then he will be able to obtain fairly reliable diagnostic and therapeutic results by simply duplicating the conditions that experience has proved to be best in certain cases; but for accurate research the induction coil will be certainly superseded in the near future by Leduc's mechanical interrupter.

The current used to excite a faradic coil may be obtained from a dry cell, a wet cell, an accumulator, or from the mains. Portable coils are usually fitted in a wooden box with a drawer for the electrodes and a separate compartment containing one or more dry cells. Bichromate cells are often used to excite medical coils and are very satisfactory when portability is not a consideration. Fig. 1 illustrates a portable coil that can be worked directly from the house mains; it is provided with an incandescent lamp, in series with the primary coil, in order to limit the primary current to the correct amount. The strength of the secondary current is regulated by sliding the secondary (sledge) coil over the primary.

### The Nature of Induced Currents.

The current, from the dry cell or other source, flowing through the primary circuit is automatically made and broken by the Neef's hammer somewhere about 100 times per second. Every time it is broken a fresh current is induced in the secondary coil, and every time it is made another fresh current is induced in the secondary coil, but this flows in a direction opposite to, and (for reasons given on page 64 of The Hospital for April 20, 1912) is weaker than the induced current at break. Thus, we see that the secondary current is really an alternating current, changing its direction perhaps 100 times per second, and much stronger in one direction than the other.

### The Effect of Induced Currents.

The physiological effects produced by induced currents from medical coils have been investigated by Lewis Jones, Leduc, and others, and, thanks to their researches, we now have a very good idea of the factors that influence motor and sensory response to these currents.

The *sensory* effects depend almost entirely upon the *duration* of the individual make and break discharges. The longer the discharges last the more painful will be the sensation produced, and *vice versâ*. If each discharge persists no longer than one-four-hundredth of a second the current will be practically painless, but if the individual discharges last as long as one-two-hundredth of a second the current will give rise to an unpleasant stinging sensation. The *rate* of interruption seems to have no influence on sensory effects, provided the individual discharges are short.

*Motor effects*, on the other hand, vary with the *rate* of interruption and with the proportion of *time* during which the current is flowing to the intervals between the discharges. Leduc found that with a given current the maximum contraction of a muscle could be produced only when the interruptions numbered 100 per second, each impulse lasted one-thousandth of a second, and the intervals between the impulses lasted nine-thousandths of a second.

The desideratum of a good coil is that it should produce a maximum of motor response with a minimum of sensory effect, and this can best be attained by using a coil with low self-induction—*i.e.*, a coil built with a short secondary and a small iron core. With such a coil the individual discharges are short, the intervals are long, and the difference between the strength of the make and break discharges is not so marked. In other words, we require a type of induction-coil discharge for faradisation that is the exact opposite of what we were at such pains to obtain for $x$-ray work.

### Sinusoidal Currents.

Sinusoidal currents are alternating currents furnished by a dynamo or by a motor transformer. They give rise to all the so-called faradic phenomena, but they differ from induction-coil currents in that they are perfectly smooth and that their discharges are of equal strength in either direction. These currents are termed *sinusoidal* because, if their gradual rise and fall be plotted out on paper, the resulting curve will represent a true *sine* curve. Such a curve presents a remarkable contrast with the irregular, jumpy curve of a medical coil.

The terminals labelled "faradic" on a motor generator, such as the Pantostat, furnish a sinusoidal current very suitable for hydro-electric baths.

Alternating current from the street mains is sinusoidal in character, but should never be used for "faradisation" until it has been transformed, because of the risk of earth shock. A very suitable transformer for this purpose consists of a small static transformer, the secondary winding of which is mounted on a sledge, so that its distance from the primary can be varied. By this means the voltage of the transformed current can be very perfectly regulated. Such a transformer is often used as an additional safeguard in connection with a motor transformer for sinusoidal baths.

### Leduc Currents.

Dr. Leduc, of Nantes, has shown that a rapidly interrupted continuous (galvanic) current will give rise to all the physiological phenomena usually associated with faradic currents. The apparatus required are a special mechanical interrupter and a galvanic battery of sufficient voltage to overcome the resistance of the human body. Leduc's pattern of interrupter is so designed that the number of impulses per second, the duration of each impulse, and the interval between them can be varied at will, while the voltage can be regulated by means of a rheostat and the exact dosage checked by a milliampere-meter.

(*To be continued.*)

## Figures and Tables

**Figure f1:**